# MicroRNA profiling of subcutaneous adipose tissue in periparturient dairy cows at high or moderate body condition

**DOI:** 10.1038/s41598-022-18956-5

**Published:** 2022-08-30

**Authors:** Hassan Sadri, Morteza Hosseini Ghaffari, Nares Trakooljul, Fabrizio Ceciliani, Helga Sauerwein

**Affiliations:** 1 Department of Clinical Science, Faculty of Veterinary Medicine, University of Tabriz, Tabriz, 5166616471 Iran; 2 Institute of Animal Science, Physiology Unit, University of Bonn, 53115 Bonn, Germany; 3 Research Institute for Farm Animal Biology (FBN), Institute of Genome Biology, Genomics Unit, 18196 Dummerstorf, Germany; 4 Dipartimento Di Medicina Veterinaria E Scienze Animali, Università Degli Studi Di Milano, 26900 Lodi, Italy

**Keywords:** Physiology, Metabolism, Fat metabolism

## Abstract

A growing body of evidence shows that microRNA (miRNA), play important roles in regulating adipose tissue (AT) physiology and function. The objective was to characterize the AT miRNA profile in over-conditioned (HBCS, *n* = 19) versus moderate-conditioned (MBCS, *n* = 19) periparturient dairy cows. Tail-head subcutaneous AT biopsied on d -49 and 21 relative to parturition were used for miRNA sequencing. The miR-486 was the most significant miRNA among the upregulated miRNA on d -49, which might be related to more pronounced changes in lipogenesis and altered insulin sensitivity in AT of HBCS cows at dry-off. Comparing HBCS to MBCS on d 21, 23 miRNA were downregulated and 20 were upregulated. The predicted targets of upregulated differentially expressed (DE)-miRNA on d 21 were enriched in different pathways, including pathways related to lysosomes and peroxisomes. The predicted targets of downregulated DE-miRNA on d 21 were enriched in various pathways, including epidermal growth factor receptor, insulin resistance, hypoxia-inducible factor 1 signaling pathway, and autophagy. The results showed that over-conditioning was associated with changes in SCAT miRNA profile mainly on d 21, of which most were downregulated. The enriched pathways may participate in over-conditioning-associated metabolic challenges during early lactation.

## Introduction

Body condition score (BCS) at calving is one of the most important factors affecting performance^1,2^, postpartum dry matter intake^3,4^ and BCS loss^5–7^, reproduction^8^, and metabolic disorders^9,10^ in periparturient dairy cows. Over-conditioned cows are more prone to have difficulties at and after parturition due to suboptimal transition from pregnancy to lactation, likely resulting in reduced productivity, economic losses, and decreasing welfare.

Adipose tissue (AT), the main lipid storage depot in the body, is a highly responsive endocrine organ that influences and interacts with metabolic homeostasis with an essential role in the successful establishment and support of lactation^11,12^. During the periparturient period, fatty acids (FA) from AT are released into the circulation under tight hormonal and neural control^13,14^ to be used as an energy source in hepatic and extrahepatic tissues^15^, causing an AT metabolism shifts towards a catabolic state^16^.

MicroRNA (miRNA), a class of small non-coding RNA, regulate gene expression through either transcriptional degradation or translational repression. There is a growing body of evidence that miRNA play a crucial role in regulating key signaling pathways in AT that control a range of processes, including adipogenesis, adipocyte differentiation, lipid metabolism, glucose homeostasis, insulin resistance, inflammation, and other metabolic and endocrine functions^17–19^. Interestingly, AT is an important source of circulating miRNA, recently described as a novel form of adipokines^20^, which may regulate gene expression and potentially function in other tissues^21^. Given the potential role of miRNA in obesity and related metabolic abnormalities, several studies have examined miRNA expression profiles in obese and lean white AT (WAT) from humans^22–24^.

The degree of variation in miRNA expression in human WAT is smaller than that of mRNA, and expression of many differentially expressed (DE)-miRNA is downregulated in obese WAT compared with lean WAT^25,26^. Vailati-Riboni et al*.* found that prepartum BCS and feeding management had a significant effect on the expression of some selected miRNA related to FA metabolism and inflammation in the subcutaneous AT (SCAT) of dairy cows during the peripartum period^27^. To the best of our knowledge, the expression pattern of miRNA in AT of periparturient dairy cows in an over-conditioned state remains still elusive.

In the present study, using small RNA sequencing approaches, we aimed to characterise the SCAT miRNA expression signatures and regulatory networks associated with over-conditioning throughout the transition period in dairy cows. We used an experimental model for dairy cows to investigate high (HBCS) versus moderate body condition (MBCS), in which cows received different diets during late lactation to reach the targeted differences in BCS and backfat thickness (BFT; HBCS: BCS > 3.75, BFT > 1.4 cm; MBCS: BCS < 3.5, BFT < 1.2 cm) until dry-off.

## Results

### Clinical health events

A summary of clinical health events during the first 6 weeks of lactation is provided in Supplemental Table S1. In the present study, the number of animals was too low to make valid comparisons of disease incidence. All cows enrolled in the study (*n* = 40) were free of disease, including mastitis. Two cows (one cow from each group) that failed to complete the sampling schedule were therefore excluded, and data of 38 cows were used. No clinical health events occurred before calving. Mastitis was the most common of clinical ailments postpartum. There were only numerical differences between HBCS and MBCS groups in the number of cows affected by clinical events (ketosis and milk fever).

### miRNA sequencing data

We generated 307 million raw reads with a mean of 27.9 ± 7.2 M reads per sample. On average, 90.3 ± 9.1% of raw reads passed filters of quality trimming (QScore < 20) and adapter-like sequence removal. Of these, a mean of 15.85 ± 5.2 M reads accounting for 63.3 ± 13.4% were mapped to bovine specific miRNA (bta-mir/miR). We detected a total of 744 sequences mapped to bta-mir/miR with at least 10 reads in at least one sample. We further filtered and used only 525 bta-mir/miR that were present in all samples for differential expression analysis.

### Differentially expressed-miRNA

**Figure 1:**
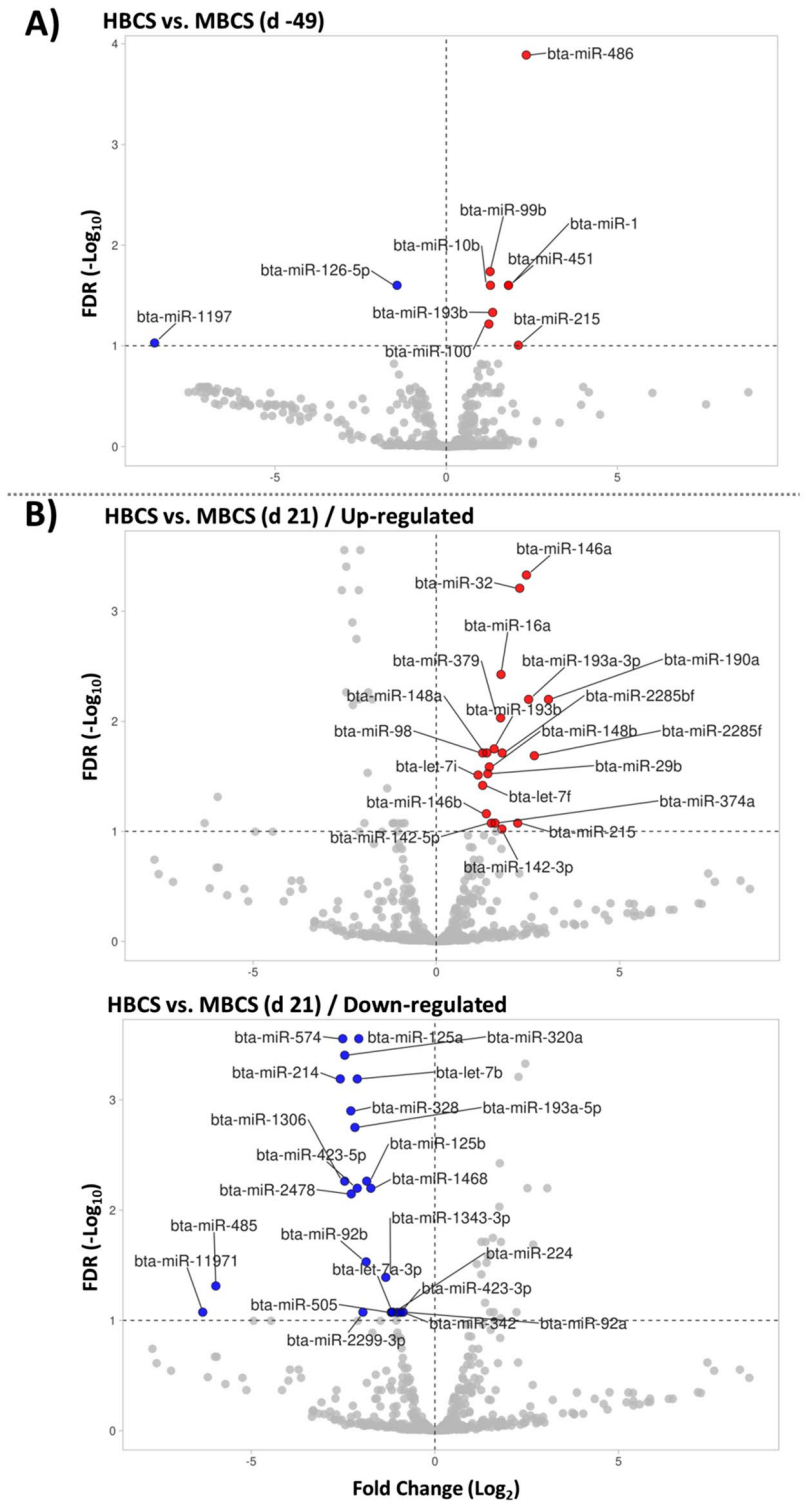
Volcano plot visualizing subcutaneous adipose tissue miRNA that were differentially expressed between high-conditioned (HBCS) and moderate-conditioned (MBCS) dairy cows on (**A**) d -49 and (**B**) 21 relative to calving. The horizontal line represents the negative logarithm of the adjusted *P*-value (FDR = 0.10). Red points show the upregulated expressed miRNA in HBCS versus MBCS cows and blue points show the downregulated miRNA.

Comparing HBCS to MBCS on d -49, 10 miRNA were differentially expressed (FDR < 0.10), including 2 downregulated and 8 upregulated miRNA (Fig. 1A). Comparing HBCS to MBCS on d 21, 43 miRNA were differentially expressed (FDR < 0.10), including 23 downregulated and 20 upregulated miRNA (Fig. 1B).

### Target prediction of DE-miRNA and KEGG pathway enrichment analyses

Target prediction and KEGG pathway enrichment analysis of up- and downregulated miRNA were performed separately. The miRNA-mRNA networks were established using the miRNet database for better visualisation, as presented in Supplemental Figs. S1–S3. The target genes of two upregulated and one downregulated DE-miRNA on d-49 (HBCS vs. MBCS) were predicted by miRNet. However, six miRNA were not mapped to the miRNet interaction database. A total of 128 and 9 genes were identified as potential targets of the upregulated (Supplemental Fig. S1A) and downregulated (Supplemental Fig. S1B) DE-miRNA on d -49, respectively. The KEGG pathway enrichment analysis revealed that the predicted targets of upregulated and downregulated DE-miRNA on d -49 were significantly enriched (FDR < 0.05) in the lysosome and spliceosome pathways.

The target genes of 15 upregulated and 23 downregulated DE-miRNA on d 21 (HBCS vs MBCS) were predicted by miRNet. However, five miRNA were not mapped to the miRNet interaction database. A total of 242 and 2046 genes were identified as potential targets of the upregulated and downregulated DE-miRNA on d 21, respectively. Among the upregulated DE-miRNA, target interactions among bta-mir-193b and bta-mir-193a-3p with at least 2 target interactions were identified, and the resulting miRNA-target interactions as a network are depicted in Supplemental Fig. S2.

**Figure 2:**
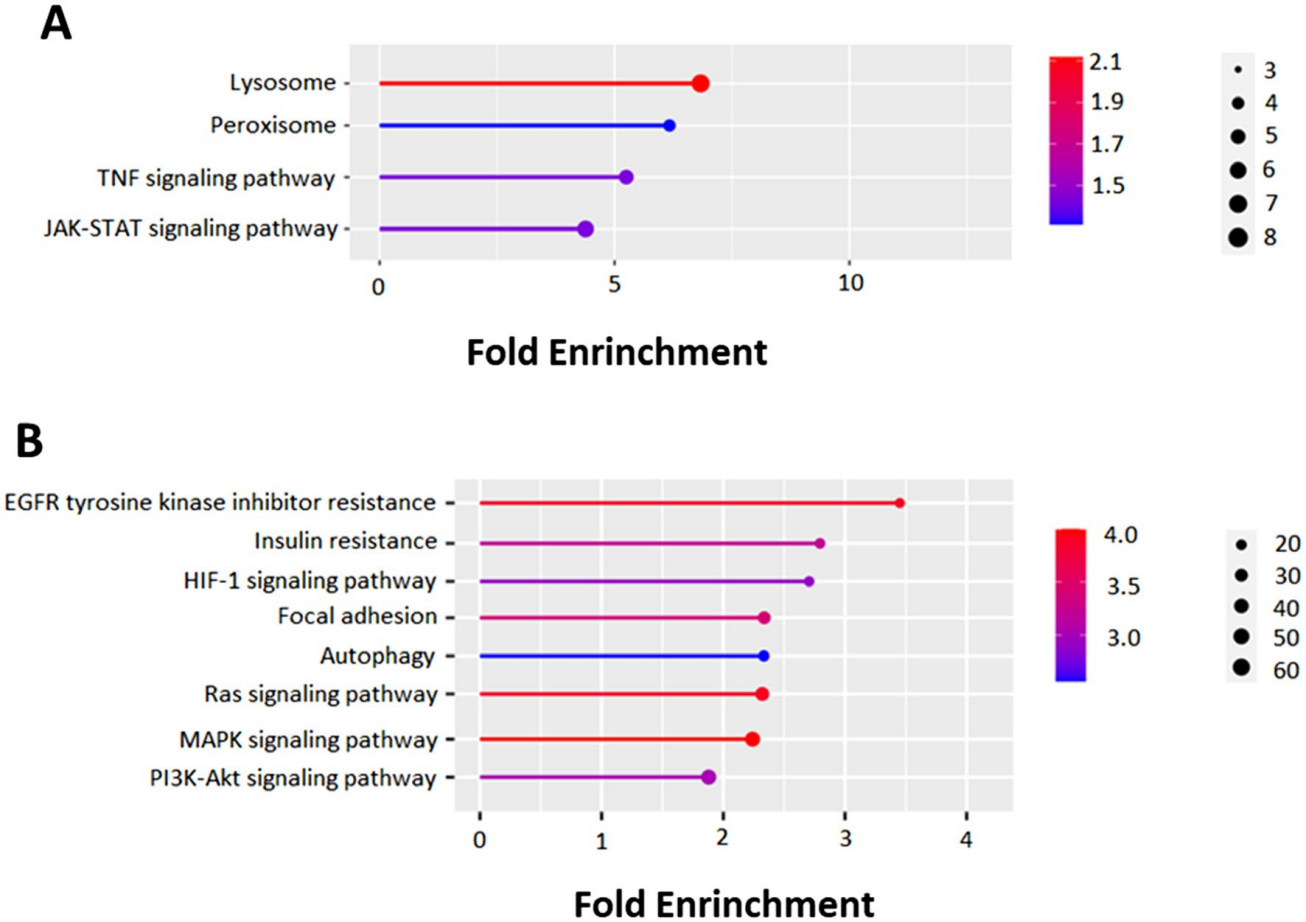
Pathway enrichment analysis of the target genes of upregulated (**A**) and downregulated (**B**) differentially expressed miRNA in subcutaneous adipose tissue of high-conditioned versus moderate-conditioned dairy cows on d 21 relative to calving annotated in Kyoto Encyclopedia of Genes and Genomes (KEGG) pathway using ShinyGO 0.76. The FDR is calculated based on the nominal P-value from the hypergeometric test. Fold Enrichment is defined as the percentage of genes in the uploaded list belonging to a pathway, divided by the corresponding percentage in the background.

The KEGG pathway enrichment analysis revealed that the predicted targets of upregulated DE-miRNA on d 21 were enriched in various pathways, including lysosome, peroxisome, tumor necrosis factor (TNF) signaling pathway, and the Janus kinase/signal transducer and activator of transcription (JAK-STAT) signaling pathway (Fig. 2A). The list of genes involved in selected relevant enriched pathways is provided in Supplemental Table S2. Target interactions among the 23 downregulated DE-miRNA were identified, and the resulting miRNA-target interaction as a network is illustrated in Supplemental Fig. S3. The list of potential target genes of downregulated DE-miRNA is given in Supplemental Excel File S1.

The top 20 KEGG pathway enrichment analysis revealed that the predicted targets of downregulated DE-miRNA on d 21 were enriched in various pathways, including epidermal growth factor receptor (EGFR) tyrosine kinase inhibitor resistance, insulin resistance, hypoxia-inducible factor 1 (HIF-1) signaling pathway, focal adhesion, autophagy, rat sarcoma (RAS) signaling pathway, mitogen-activated protein kinase (MAPK) signaling pathway, and phosphatidylinositol 3'-kinase(PI3K)/protein kinase B (AKT) signaling pathway (Fig. 2B). The list of genes involved in selected relevant enriched pathways is reported in Supplemental Table S3.

### Quantitative real-time PCR (qPCR) validation of miRNA expression

**Figure 3:**
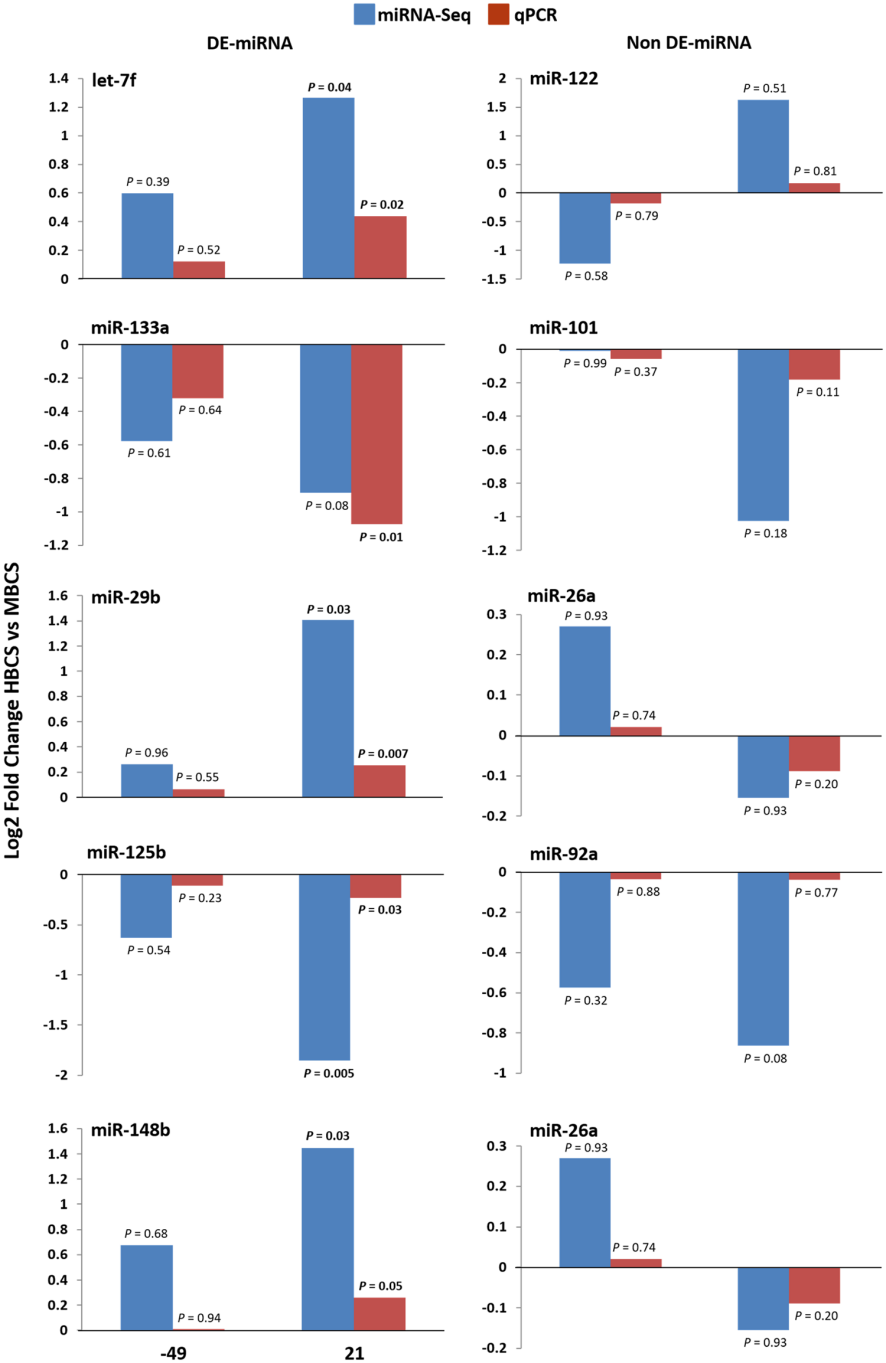
Comparison of the log2fold-change values obtained by either miRNA-Seq or RT-qPCR for five randomly selected differentially expressed (DE)-miRNA and five non DE-miRNA miRNA in high (HBCS) versus moderate-conditioned (MBCS) dairy cows on d -49 and 21 relative to calving. The y-axis represents the mean of log2fold-change value comparing HBCS versus MBCS n d -49 and 21. The level of significance was set at *P* < 0.05. The miRNA-seq results are based on pooled samples (4 pools containing each 4 animals per time point), whereas individual samples were used for the RT-qPCR validation.

The RT-qPCR was used to validate the miRNA-Seq expression results of five randomly selected DE-miRNA (let-7, miR-133a, miR-29b, miR-148b, and miR-125b) and five non DE-miRNA (miR-101, miR-122, miR-26a, miR-27b, and miR-92a) identified in the current study (Fig. 3). Expression trends of miRNA by qPCR were generally similar to the results from miRNA sequencing. Four of five miRNA (let-7, miR-29b, miR-148b, and miR-125b) were significantly DE by both miRNA-Seq and RT-qPCR methods on d 21, wherease miR-133 DE on d 21 by the method of qPCR only tended (*P* = 0.08) to be DE by miRNA-Seq method. Similar results of miRNA-Seq and qPCR were observed for non DE-miRNA (Fig. 3).

## Discussion

The present study compared HBCS to MBCS at dry-off (d -49) and early postpartum (d 21). The most differentially downregulated and upregulated miRNA in the SCAT were determined using a high-throughput small RNA sequencing and were observed on d 21. Expression trends of randomly selected DE- and non DE-miRNA in the individual samples from the same populations by qPCR were similar to the results from miRNA-sequencing, further validating the biological conclusions from the RNA-sequencing results in the current study.

The miR-486 was the most significant miRNA among the upregulated miRNA in HBCS cows compared to MBCS cows on d -49. The miRNA profiling of obese versus lean children has shown elevated concentrations of miR-486 in plasma and is associated with body mass index, percent fat mass, insulin resistance, and circulating lipids^28^. In support of this, Cui et al*.* found that circulating miR-486 was dramatically (> sixfold) augmented in obese compared with non-obese children and adults with type 2 diabetes^29^. The miR-486 might be an important mediator in glucose metabolism, as over-expression of miR-486 was implicated in accelerating human preadipocyte proliferation and glucose intolerance in C2C12 myoblast cells in vitro^29^.

Thus, the observed differences in miR-486 might be related to more pronounced changes in lipogenesis, glucose metabolism, or altered insulin sensitivity in the AT of HBCS cows compared with MBCS cows. Indeed, increased lipogenesis in HBCS cows was reflected by greater BCS and BFT. In support of this, in the companion study by Sadri et al., the mRNA abundance of key genes related to lipogenesis such as *acetyl-CoA carboxylase, FA synthase*, and *glycerol-3-phosphate acyltransferase* in HBCS cows was greater than those of MBCS cows on d -49^30^. The miR-486 is considered an adipose-derived circulating miRNA^17^ and in a companion study^31^, it was identified as one of the most abundant miRNA in serum of dairy cows on d -49, but did not differ between HBCS and MBCS cows. However, Ylioja et al*.* found that colostrum miR-486 was less abundant in cows with high BCS (≥ 4.0) compared with cows with moderate BCS (2.75–3.50) at calving, which has been linked with altered glucose metabolism^32^.

Pathway analysis showed that the predicted targets of upregulated DE-miRNA (i.e. miR-486 and miR-193b) on d -49 were enriched in lysosome-related pathway. The importance of lysosomal function in obesity-associated lipid metabolism in AT macrophages^33^ and inflammation in adipocyte^34–36^ as well as lipid-related disorders^37^ was shown in human medicine. Luo et al. found a reduced abundance of acidified lysosomes in pre-adipocytes as a trigger for inflammation in diet-induced obesity in both human and mice^35^. In the current study, the increase in the BCS and BFT of cows in the HBCS group was also achieved through feeding a high-energy diet from 15 weeks ante partum until dry-off (d -49); however, it is not clear whether or not the observed upregulation in miR-486 and miR-193b on d -49 and potential changes in the lysosome pathway were also associated with changes in metabolic processes at the AT level, such as inflammation.

Pathway analysis revealed that the predicted targets of downregulated DE-miRNA on d -49 were enriched in the spliceosome pathway. The spliceosome is assembled from small nuclear RNA (snRNA) and associated proteins and removes introns from pre-mRNA transcripts (also known as splicing; Matera and Wang)^38^. The action of the spliceosome component on multiple key genes contributing to adipogenesis and lipogenesis was demonstrated in obese individuals with insulin resistance and type 2 diabetes^39^, which seems to be in line with increasing fat deposition, BFT, and BCS of HBCS cows on d -49 in the current study.

Pathway analysis showed that the predicted targets of upregulated DE-miRNA on d 21 (i.e., miR-193a-3p and miR-193b), similar to d -49, were enriched in a pathway related to lysosome, further suggesting that the lysosome might be associated with over-conditioning. Besides pathways related to lysosome, the predicted targets of upregulated DE-miRNA on d 21 were also enriched in peroxisome-related functions. Peroxisomes play important roles in lipid metabolism, such as regulating adipogenesis, adipocyte FA oxidation, and biosynthesis of specific lipids, including ether lipids and cholesterol^40,41^. The physiological importance of mitochondrial or peroxisomal β-oxidation in WAT, in general, has less been appreciated as oxidation rates are relatively low as compared with other tissues like skeletal muscle^42,43^. However, some human and rodent studies show that FA oxidation in WAT plays an important role in metabolic homeostasis^44,45^, contributing effectively to buffering tissue FA levels and preventing metabolic stress and insulin resistance in AT^46,47^.

Thus, it is speculated that the observed upregulation of miR-193a-3p and miR-193b might be associated with repression in the expression of peroxisomal FA β-oxidation related genes in the AT of HBCS cows compared with MBCS cows. Moreover, over-expression of miRNA that are dysregulated in obese human WAT resulted in stimulation of lipolysis by some miRNA, including miR-193b^48^. As reported in the companion study by Schuh et al., HBCS cows had greater serum concentration of FA and thus more lipolysis than MBCS cows postpartum^7^. This is in agreement with the upregulation of miR-193b in HBCS cows observed in the present study.

The predicted targets of downregulated DE-miRNA on d 21 were enriched in some pathways that are in line with our previous observations of performance and metabolism in the same animals^7^: HBCS cows were metabolically challenged during early lactation due to a more severe negative energy balance and intense mobilization of body fat, associated with reduced early lactation dry matter intake. In addition to the known lipolysis process mediated by hormone-sensitive lipase (HSL) and adipose triglyceride lipase (ATGL), the breakdown of lipids can also take place through lipophagy^49^, a type of selective autophagy that targets intracellular lipid droplets and is considered an essential mechanism for maintaining homeostasis of lipid droplets^50,51^. As reported previously from this animal experiment^30^, the mRNA abundance of genes related to lipolysis, including *HSL* and *ATGL* in SCAT, did not differ between HBCS and MBCS cows on d 21, suggesting a possible role for the lipophagy-dependent pathway in the increased mobilization of body fat in HBCS cows.

Insulin resistance was another significant pathway that was enriched by down-regulated DE-miRNA on d 21. Periparturient dairy cows exhibit altered insulin sensitivity (**IS**) during the adaptation to lactation, a phenomenon that has been ascribed to multiple factors^52^. As reported in the companion study by Schuh et al., HBCS cows had greater insulin concentrations than MBCS cows, accompanied by greater glucose concentrations which might reflect reduced IS in HBCS cows^7^. It has been shown that IS decreases with increased BCS in dry or late lactating cows^53^. In the AT, insulin acts as an anti-lipolytic hormone; thus, insulin resistance might have led to the further lipid mobilisation from AT in HBCS cows.

Laboratory animal studies have found that AT becomes hypoxic in obesity^54–56^, which plays a crucial role in developing obesity-associated metabolic disorders, such as inflammation and insulin resistance^57^. In the current study, the predicted targets of downregulated DE-miRNA on d 21 were enriched in the HIF-1 signaling pathway, likely proposing a potential association of SCAT HIF with over-conditioning and its potential involvement in decreasing IS with increased BCS. The HIF-1 is a transcription factor that acts as a master regulator of oxygen homeostasis. Reduced blood flow to WAT, increased size of adipocytes reaching in obese subjects a diameter larger than 150–200 µm (thus exceeding the normal capacity of oxygen diffusion through the tissue), or enhanced oxygen consumption by adipocytes or inflammatory cells infiltrated into the obese microenvironment are considered as potential contributors to the onset of AT hypoxia in obesity^58^. Increased HIF-1α-positive cells in histological sections of SCAT were positively correlated with adipocyte size and BCS in non-pregnant and non-lactating cows^59^. We thus hypothesize that SCAT from HBCS cows might have undergone hypoxia, which warrants further investigations.

The EGFR is a transmembrane tyrosine kinase receptor and a crucial component of cell signal pathways, participating in the regulation of cellular homeostasis. The EGFR has gained special attention in human medicine, given its associations with the development of lung cancer^59,60^. The EGFR has three main downstream signaling pathways: (1) RAS/rapidly accelerated fibrosarcoma (RAF)/MAPK pathway; (2) PI3K/AKT pathway, and (3) JAK/STAT pathway, which stimulate mitosis, leading to cell proliferation and inhibition of apoptosis^61^. Interestingly, the EGFR and its first two main downstream signaling pathways were enriched by downregulated DE-miRNA on d 21, whereas the JAK/STAT pathway was enriched by upregulated DE-miRNA on d 21.

Studies regarding the EGFR signaling pathway in the context of metabolic regulation in humans and laboratory animals are controversial. Still, most studies have shown that alteration of the EGFR and its downstream signaling pathways impact various aspects of AT metabolism, including insulin sensitivity, modulation of lipid stores, and glucose homeostasis^62^. Potential changes in the EGFR and its downstream signaling pathways and the enriched metabolic pathways on d 21 that were discussed above suggest the involvement of DE-miRNA in shifting AT metabolism towards catabolism, possibly also modulating insulin sensitivity glucose metabolism, lipolysis, and inflammation.

## Conclusion

To our knowledge, this is the first report about miRNA profiles of SCAT in over- versus moderate-conditioned dairy cows during the periparturient period. Over-conditioning was associated with changes in the profile of the SCAT miRNA mainly on d 21, and the majority of these DE-miRNA was downregulated in HBCS cows compared with MBCS cows. Comparing HBCS to MBCS on d -49, miR-486 was the most significant miRNA among the upregulated miRNA, which may be related to more pronounced changes in insulin resistance, glucose utilization, and lipogenesis in SCAT of over-conditioned cows compared with cows of moderate BCS. The predicted targets of DE-miRNA on d 21 were enriched in various pathways, including EGFR, insulin resistance, HIF-1 signaling, and autophagy, which highlight the involvement of miRNA in the shift of AT metabolism towards catabolism, intense mobilization of body fat, and over-conditioning-associated metabolic challenges. Extending future work from pooled to individual samples may identify other miRNA in this context and boost the characterization of inter-individual variation.

## Materials and methods

### Animals, treatments, and sample collection

The experiment was carried out at the experimental station of the Educational and Research Centre for Animal Husbandry, Hofgut Neumühle, Muenchweiler a.d. Alsenz, Germany. All study protocols were designed and conducted in compliance with the European Union Guidelines concerning the protection of experimental animals, with approval by the local authority for animal welfare affairs (Landesuntersuchungsamt Rheinland-Pfalz, Koblenz, Germany [G 14–20-071]). The study is reported according to the ARRIVE guidelines.

The animals were part of a trial to establish an experimental model of high versus moderate body tissue mobilization around calving. Details of the experimental design, together with performance data and classical metabolites and metabolic hormones, have already been reported^7^. In brief, fifteen weeks before calving, 38 multiparous German Holstein cows (average parity 2.9 ± 0.3; mean ± SEM) were allocated to either a moderate-conditioned (MBCS; *n* = 19) or high-conditioned group (HBCS; *n* = 19). In order to reach the targeted differences in BCS (a 5-point BCS system^63^) and BFT in the experimental groups [HBCS: BCS > 3.75 (3.82 ± 0.33) and BFT > 1.4 cm (2.36 ± 0.35); means ± SD; MBCS: BCS < 3.5 (3.02 ± 0.24) and BFT < 1.2 cm (0.92 ± 0.21)] until dry-off (week 7 ante partum), HBCS cows received a more energy-dense ration (7.2 MJ NE_L_/kg DM) than the MBCS cows (6.8 MJ NE_L_/kg DM) during late lactation (from week 15 to 7 before the anticipated calving date). The two groups received identical diets during the dry period and subsequent lactation. Cows were offered ad libitum intake of a total mixed ration (TMR) consisting of 63% roughage and 37% concentrate in the high-energy diet, or 74% roughage, and 26% concentrate in the low-energy diet. The diets were formulated to meet or exceed the nutritional requirements of dairy cows according to the recommendation of the Society of Nutrition Physiology^64^. Backfat tickness was assessed in the sacral region using ultra-sonography (AGROSCAN L, ALR500, 5 MHz, linear-array transducer; Echo Control Medical,Angoulême, France).

The SCAT samples were collected from alternate sides of the tail head region with a scalpel through an incision of 1 cm width on days (d) -49 and 21 relative to calving under local anesthesia (procaine hydrochloride, 20 mg/mL, 9 mL per biopsy; Richter Pharma AG, Wels, Austria) while the animals were sedated (Xylazine i.v., 20 mg/mL, 0.1 mL/100 kg BW; CP-Pharma Handels GmbH, Burgdorf, Germany) and fixed in a headlock. Immediately after sampling, incisions were closed with a sterile needle and absorbable suture (Spool suture PGA, USP 1, EP 4, LOT 15B27, Henry Schein U.K. Holdings Ltd, Gillingham, UK). To prevent infection and for analgesia, respectively, oxytetracycline hydrochloride was applied to the skin (25 mg/mL, EngemycinTM, MSD Animal Health Innovation GmbH, Schwabenheim an der Selz, Germany) and a ketoprofen injection (100 mg/mL, 3 mL/100 kg BW; Streuli Pharma AG, Uznach, Switzerland) was given. Tissue samples were rinsed with 0.9% NaCl to remove any blood contamination, immediately snap-frozen in liquid nitrogen, and stored at − 80 °C until analysis.

### miRNA extraction, library generation, and sequencing

Total RNA enriched for small RNA, including miRNA was isolated from SCAT samples using the Qiagen miRNeasy kit (Qiagen, Hilden, Germany) according to the manufacturer's recommendation. The quality and quantity of small RNA were assessed using an Agilent Small RNA kit on a 2100 Bioanalyzer (Agilent Technologies, Waldbronn, Germany). Samples were then pooled (4 pools with 4 individuals per group and day). Pooling of samples was performed at the RNA level by mixing randomly selected RNA samples extracted from independent biological samples from the same group and equal amounts of RNA from each vole, before library preparation.

Libraries were prepared with NEXTFLEX Small RNA-seq v3 Kits with UDIs for Illumina (PerkinElmer) according to the manufacturer’s recommendations. The quality of the libraries was assessed using the Highly Sensitive DNA assay and a 2100 Bioanalyzer (Agilent). A total of 12 multiplexed libraries were parallel sequenced for single-end of 50 bp using the rapid-run mode of the Illumina HiSeq 2500 system by the sequencing facility of the Genome Biology Institute, FBN, Dummerstorf, Germany.

### Detection and expression profiling of miRNA

The base call files from the sequencing run were de-multiplexed and converted into FASTQ files using the bcl2fastq2 conversion software, version 2.20 (Illumina). The FASTQ-formatted sequence data were quality-checked using FastQC version 0.11.9^65^. The sequence data were pre-processed using Trim Galore (v0.6.6) to remove low-quality and adapter-like sequences (https://github.com/FelixKrueger/TrimGalore). The detection and expression profiling of miRNA was performed using the miRDeep2 software package^66,67^. Briefly, the pre-processed high-quality reads [by trimming low-quality reads (mean Q-score < 20) and filtering too short or too long reads (> 26 or < 16 nucleotides)] were aligned to the bovine reference genome assembly, ARS-UCD1.2 using mirDeep2. Known and novel miRNA in the sequencing samples were detected by the miRDeep2 algorithms based on positional alignment, secondary RNA structure, entropy, biogenesis-based model, and the reference of bovine miRNA and conserved miRNA cross-species (human and mouse) from miRBase 22 release. Novel miRNA were excluded from further analysis.

### miRNA target prediction and pathway analysis

We performed a univariate analysis with a volcano plot generated by VolcaNoseR^68^ to determine the DE-miRNA (FDR < 0.10) between the HBCS and MBCS groups on d -49 and 21. The potential target genes of the DE-miRNA were predicted using miRNet 2.0 (http://www.mirnet.ca/), which is a web-based platform that integrated data from eleven different miRNA databases (TarBase, miRTarBase, miRecords, miRanda, miR2Disease, HMDD, PhenomiR, SM2miR, PharmacomiR, EpimiR and starBase) and displayed the association in a visual network^69^.

Then, the miRNA target genes were annotated in the Kyoto Encyclopedia of Genes and Genomes (KEGG) pathway using the ShinyGO 0.76 online web tool (http://bioinformatics.sdstate.edu/go; Ge et al.^70^). Enrichment analysis was based on hypergeometric distribution followed by FDR correction (< 0.05). The FDR is calculated based on the nominal P-value from the hypergeometric test. Fold Enrichment is the percentage of genes in the uploaded list belonging to a pathway, divided by the corresponding percentage in the background^70^. Background gene-sets were all protein-coding genes in the genome.

### Validation of candidate miRNA using RT-qPCR

To validate the results of sequencing, the expression of five randomly selected differentially expressed (DE)-miRNA and five non DE-miRNA was quantified in sequenced samples and was extended to individual samples by reverse transcription (RT) qPCR using Taqman miRNA assays (Thermo Fisher Scientific, Darmstadt, Germany). The probes included hsa-let-7f. (Assay ID: 000382), hsa-miR-133a (002246), hsa-miR-29b (000413), hsa-miR-148b (000471), hsa-miR-125b (000449), hsa-miR-1 (002222), hsa-miR-122 (002245), hsa-miR-26a (000405), hsa-miR-27b (000409), and hsa-miR-92a (000431). The RT-qPCR analysis was performed using the BioMark HD 192 × 24 system (Fluidigm). Reverse Transcription (RT) was performed using the Applied Biosystems Megaplex (Thermo Fisher Scientific, Dreieich, Germany) according to the manufacturer’s protocol.

A pre-amplification of cDNA was performed to create specific target amplified reactions. The pre-amplification reaction solution was prepared in a total volume of 5 μL using the Applied Biosystems Preamp Master Mix (Thermo Fisher Scientific): 2.5 μL of 2 × TaqMan Preamp Master Mix, 0.5 μL 10 × MegaPlex™ PreAmp Primers, and 2.0 μL of cDNA. Thermal cycling conditions for reactions were as follows: activation of the polymerase at 95 °C for 10 min, followed by 17 cycles of denaturation at 95 °C for 15 s and annealing at 55 °C for 2 min and an extension at 72 °C for 2 min. The pre-amplified cDNA was stored at -20 °C and was freshly diluted 1:10 before using 45 µL of DNA Suspension Buffer (10 mM Tris, pH 8.0, 0.1 mM EDTA) to 5 µL of each sample.

Multiplex RT-qPCR was conducted using BioMark 192 × 24 Gene Expression Dynamic Array chips (Fluidigm). The 10 × assay mix was prepared in a total volume of 3 μL (final concentration: primers 9 µM; probes 2 µM) comprising 1.5 µL of each 20 × TaqMan Gene Expression Assay (Life Technologies) and 1.5 µL of 2 × Assay Loading Reagent (Fluidigm). The sample mix was prepared in a total volume of 3 μL comprising 1.5 μL of 2 × TaqMan FAST Universal PCR Master Mix (Life Technologies), 0.15 µL of 20 × GE Sample Loading Reagent (Fluidigm), and 1.35 μL of preamplified and 1:10 diluted cDNA. All reactions were performed in duplicates. The RT-qPCR was performed using the BioMark HD: GE 192 × 24 v1 protocol.

The qPCR reactions were validated with the Fluidigm Real-Time PCR Analysis Software version 4.7.1 (Fluidigm). To determine the most stably expressed miRNA for RT-qPCR data normalization, the quantification cycle (Cq) values were exported, and expression stabilities were calculated using the web-based tool RefFinder^71^. RefFinder integrates the currently available major computational programs (geNorm, Normfinder, BestKeeper, and the comparative Delta-Ct method) to compare and rank the tested candidate reference genes. The expression stabilities calculated according to different algorithms were largely concordant. Two miRNA, miR-101 and miR-26a (with a geNorm M-value below 0.5), were determined as the most stable reference miRNA. The geometric mean of the relative quantities of the reference miRNA was used for normalization.

In order to compare the result of RT-qPCR and miRNA-Seq, log2 fold-change was calculated by comparing relative abundance of the miRNA between HBCS and MBCS on d -49 and 21. The t-test was used to compare means between HBCS and MBCS groups within time-points. The level of significance was set at *P* < 0.05.

## Supplementary Information

Supplementary Information 1.

Supplementary Information 2.

Supplementary Information 3.

## Data Availability

The data that support the findings of this study are available from the corresponding author, [H. Sauerwein], upon reasonable request.

## References

[1] Berry DP, Buckley F, Dillon P (2007). Body condition score and live-weight effects on milk production in Irish Holstein-Friesian dairy cows. Animal.

[2] Roche JR, Lee JM, Macdonald KA, Berry DP (2007). Relationships among body condition score, body weight, and milk production variables in pasture-based dairy cows. J. Dairy Sci..

[3] Garnsworthy PC, Jones GP (1987). The influence of body condition at calving and dietary-protein supply on voluntary food intake and performance in dairy cows. Anim. Prod..

[4] Roche JR (2008). Neuroendocrine and physiological regulation of intake, with particular reference to domesticated ruminant animals. Nutr. Res. Rev..

[5] Roche JR, Berry DP, Lee JM, Macdonald KA, Boston RC (2007). Describing the body condition score change between successive calvings: A novel strategy generalizable to diverse cohorts. J. Dairy Sci..

[6] Gärtner T, Gernand E, Gottschalk J, Donat K (2019). Relationships between body condition, body condition loss, and serum metabolites during the transition period in primiparous and multiparous cows. J. Dairy Sci..

[7] Schuh K (2019). Comparison of performance and metabolism from late pregnancy to early lactation in dairy cows with elevated v. normal body condition at dry-off. Animal.

[8] Buckley F, O’Sullivan K, Mee JF, Evans RD, Dillon P (2003). Relationships among milk yield, body condition, cow weight, and reproduction in spring-calved Holstein-Friesians. J. Dairy Sci..

[9] Gillund P, Reksen O, Grohn YT, Karlberg K (2001). Body condition related to ketosis and reproductive performance in Norwegian dairy cows. J. Dairy Sci..

[10] Roche JR, Berry DP (2006). Periparturient climatic, animal, and management factors influencing the incidence of milk fever in grazing systems. J. Dairy Sci..

[11] McNamara JP, Huber K (2018). Metabolic and endocrine role of adipose tissue during lactation. Annu. Rev. Anim. Biosci..

[12] Häussler S, Sadri H, Ghaffari MH, Sauerwein H (2022). Symposium review: Adipose tissue endocrinology in the periparturient period of dairy cows. J Dairy Sci..

[13] McNamara JP (2015). Systems biology of regulatory mechanisms of nutrient metabolism in lactation. J. Anim. Sci..

[14] Contreras GA, Strieder-Barboza C, Raphael W (2017). Adipose tissue lipolysis and remodeling during the transition period of dairy cows. J. Anim. Sci. Biotechnol..

[15] Grummer RR (1993). Etiology of lipid-related metabolic disorders in periparturient dairy cows. J. Dairy Sci..

[16] McNamara JP (1991). Regulation of adipose tissue metabolism in support of lactation. J. Dairy Sci..

[17] Icli B, Feinberg MW (2017). MicroRNAs in dysfunctional adipose tissue: cardiovascular implications. Cardiovasc. Res..

[18] Gharanei S (2020). Regulatory microRNAs in brown, brite and white adipose tissue. Cells.

[19] Heyn GS, Corrêa LH, Magalhães KG (2020). The impact of adipose tissue-derived miRNAs in metabolic syndrome, obesity, and cancer. Front. Endocrinol..

[20] Withers SB, Dewhurst T, Hammond C, Topham CH (2020). MiRNAs as novel adipokines: Obesity-related circulating MiRNAsin*fl*uence chemosensitivity in cancer patients. Non Cod. RNA..

[21] Thomou T (2017). Adipose-derived circulating miRNAs regulate gene expression in other tissues. Nature.

[22] Heneghan HM, Miller N, McAnena OJ, O’Brien T, Kerin MJ (2011). Differential miRNA expression in omental adipose tissue and in the circulation of obese patients identifies novel metabolic biomarkers. J. Clin. Endocrinol. Metab..

[23] Capobianco V (2012). miRNA and protein expression profiles of visceral adipose tissue reveal miR-141/YWHAG and miR-520e/RAB11A as two potential miRNA/protein target pairs associated with severe obesity. J. Proteome Res..

[24] Wang L (2021). MicroRNA expression profiles in the subcutaneous adipose tissues of morbidly obese Chinese women. Obes. Facts..

[25] Civelek M (2013). Genetic regulation of human adipose microRNA expression and its consequences for metabolic traits. Hum. Mol. Genet..

[26] Arner P, Kulyté A (2015). MicroRNA regulatory networks in human adipose tissue and obesity. Nat. Rev. Endocrinol..

[27] Vailati-Riboni M (2016). Body condition score and plane of nutrition prepartum affect adipose tissue transcriptome regulators of metabolism and inflammation in grazing dairy cows during the transition period. J. Dairy Sci..

[28] Prats-Puig A (2013). Changes in circulating microRNAs are associated with childhood obesity. J. Clin. Endocrinol. Metab..

[29] Cui X (2018). Change in circulating microRNA profile of obese children indicates future risk of adult diabetes. Metabolism.

[30] Sadri H, Ghaffari MH, Schuh K, Koch C, Sauerwein H (2021). Muscle metabolome and adipose tissue mRNA expression of lipid metabolism-related genes in over-conditioned dairy cows differing in serum-metabotype. Sci. Rep..

[31] Webb LA (2020). Profiling of circulating microRNA and pathway analysis in normal- versus over-conditioned dairy cows during the dry period and early lactation. J. Dairy Sci..

[32] Ylioja CM, Rolf MM, Mamedova LK, Bradford BJ (2019). Associations between body condition score at parturition and microRNA profile in colostrum of dairy cows as evaluated by paired mapping programs. J. Dairy Sci..

[33] Xu X (2013). Obesity activates a program of lysosomal-dependent lipid metabolism in adipose tissue macrophages independently of classic activation. Cell Metab..

[34] Ju L (2019). Obesity-associated inflammation triggers an autophagy-lysosomal response in adipocytes and causes degradation of perilipin 1. Cell Death Dis..

[35] Luo X (2020). Obesity induces preadipocyte C36 expression promoting inflammation via disruption of lysosomal calcium homeostasis and lysosome function. EBioMedicine.

[36] Rawnsley DR, Diwan A (2020). Lysosome impairment as a trigger for inflammation in obesity: The proof is in the fat. EBioMedicine.

[37] Cabrera-Reyes F, Parra-Ruiz C, Yuseff MI, Zanlungo S (2021). Alterations in lysosome homeostasis in lipid-related disorders: Impact on metabolic tissues and immune cells. Front. Cell Dev. Biol..

[38] Matera AG, Wang Z (2014). A day in the life of the spliceosome. Nat. Rev. Mol. Cell Biol..

[39] Sánchez-Ceinos J (2021). Impaired mRNA splicing and proteostasis in preadipocytes in obesity-related metabolic disease. Elife.

[40] Wanders RJ (2013). Metabolic functions of peroxisomes in health and disease. Biochimie.

[41] Liu J, Lu W, Shi B, Klein S, Su X (2019). Peroxisomal regulation of redox homeostasis and adipocyte metabolism. Redox Biol..

[42] Harper RD, Saggerson ED (1975). Some aspects of fatty acid oxidation in isolated fat-cell mitochondria from rat. Biochem. J..

[43] Böttcher H, Fürst P (1997). Decreased white fat cell thermogenesis in obese individuals. Int. J. Obes. Related Metab. Dis..

[44] Marcelin G, Chua S (2010). Contributions of adipocyte lipid metabolism to body fat content and implications for the treatment of obesity. Curr. Opin. Pharmacol..

[45] Kusminski CM, Scherer PE (2012). Mitochondrial dysfunction in white adipose tissue. Trends Endocrinol. Metab..

[46] Ahmadian M, Duncan RE, Sul HS (2009). The skinny on fat: lipolysis and fatty acid utilization in adipocytes. Trends Endocrinol. Metab..

[47] Torchon E, Ray R, Hulver MW, McMillan RP, Voy BH (2017). Fasting rapidly increases fatty acid oxidation in white adipose tissue of young broiler chickens. Adipocyte..

[48] Lorente-Cebrián S (2014). MicroRNAs regulate human adipocyte lipolysis: effects of miR-145 are linked to TNF-α. PLoS ONE.

[49] Singh R (2009). Autophagy regulates lipid metabolism. Nature.

[50] Schulze RJ, Sathyanarayan A, Mashek DG (2017). Breaking fat: The regulation and mechanisms of lipophagy. Biochim. Biophys. Acta Mol. Cell Biol. Lipids..

[51] Shin DW (2020). Lipophagy: Molecular mechanisms and implications in metabolic disorders. Mol. Cells..

[52] De Koster JD, Opsomer G (2013). Insulin resistance in dairy cows. Vet. Clin. North Am. Food Anim. Pract..

[53] De Koster J (2015). Insulin response of the glucose and fatty acid metabolism in dry dairy cows across a range of body condition scores. J. Dairy Sci..

[54] Hosogai N (2007). Adipose tissue hypoxia in obesity and its impact on adipocytokine dysregulation. Diabetes.

[55] Ye J, Gao Z, Yin J, He Q (2007). Hypoxia is a potential risk factor for chronic inflammation and adiponectin reduction in adipose tissue of ob/ob and dietary obese mice. Am. J. Physiol. Endocrinol. Metab..

[56] Rausch ME, Weisberg S, Vardhana P, Tortoriello DV (2008). Obesity in C57BL/6J mice is characterized by adipose tissue hypoxia and cytotoxic T-cell infiltration. Int. J. Obes. (Lond.).

[57] Ban JJ, Ruthenborg RJ, Cho KW, Kim JW (2014). Regulation of obesity and insulin resistance by hypoxia-inducible factors. Hypoxia (Auckl.).

[58] Laubenthal L (2017). Effect of increasing body condition on oxidative stress and mitochondrial biogenesis in subcutaneous adipose tissue depot of nonlactating dairy cows. J. Dairy Sci..

[59] Shah R, Lester JF (2020). Tyrosine kinase inhibitors for the treatment of EGFR mutation-positive non-small-cell lung cancer: A clash of the generations. Clin. Lung Cancer..

[60] Ikeuchi H (2022). Preclinical assessment of combination therapy of EGFR tyrosine kinase inhibitors in a highly heterogeneous tumor model. Oncogene.

[61] Huang L, Fu L (2015). Mechanisms of resistance to EGFR tyrosine kinase inhibitors. Acta. Pharm. Sin. B..

[62] Zhao M, Jung Y, Jiang Z, Svensson KJ (2020). Regulation of energy metabolism by receptor tyrosine kinase ligands. Front. Physiol..

[63] Edmonson AJ, Lean IJ, Weaver L, Farver DT, Webster GA (1989). A body condition scoring chart for Holstein dairy cows. J. Dairy Sci..

[64] GfE (German Society of Nutrition Physiology). Ausschuss für Bedarfsnormen der Gesellschaft für Ernährungsphysiologie. Nr. 8. Empfehlungen zur Energie- und Nährstoffversorgung der Milchkühe und Aufzuchtrinder (Recommendations of energy and nutrient supply for dairy cows and breeding cattle). DLG-Verlag, Frankfurt am Main, Germany (2001).

[65] Babraham Bioinformatics. FastQC: a quality control tool for high throughput sequence data. http://www.bioinformatics.babraham.ac.uk/ projects/fastqc (2018).

[66] Mackowiak SD (2011). Identification of novel and known miRNAs in deep-sequencing data with miRDeep2. Curr. Protoc. Bioinformatics..

[67] Friedländer MR, Mackowiak SD, Li N, Chen W, Rajewsky N (2012). miRDeep2 accurately identifies known and hundreds of novel microRNA genes in seven animal clades. Nucl. Acids Res..

[68] Goedhart J, Luijsterburg MS (2020). VolcaNoseR is a web app for creating, exploring, labeling and sharing volcano plots. Sci. Rep..

[69] Chang L, Zhou G, Soufan O, Xia J (2020). miRNet 2.0 - network-based visual analytics for miRNA functional analysis and systems biology. Nucl. Acids Res..

[70] Ge SX, Jung D, Yao R (2020). ShinyGO: A graphical gene-set enrichment tool for animals and plants. Bioinformatics.

[71] Xie F, Xiao P, Chen D, Xu L, Zhang B (2012). miRDeepFinder: a miRNA analysis tool for deep sequencing of plant small RNAs. Plant Mol. Biol..

