# Supplementary material for: MicroRNA profiling of subcutaneous adipose tissue in periparturient dairy cows at high or moderate body condition
Source: Sci Rep. 2022 Aug 30;12:14748. doi: 10.1038/s41598-022-18956-5 (PMC9427980; doi:10.1038/s41598-022-18956-5)
Supplement: Supplementary file 2 — Supplementary Information 2. [file 41598_2022_18956_MOESM2_ESM.pdf]

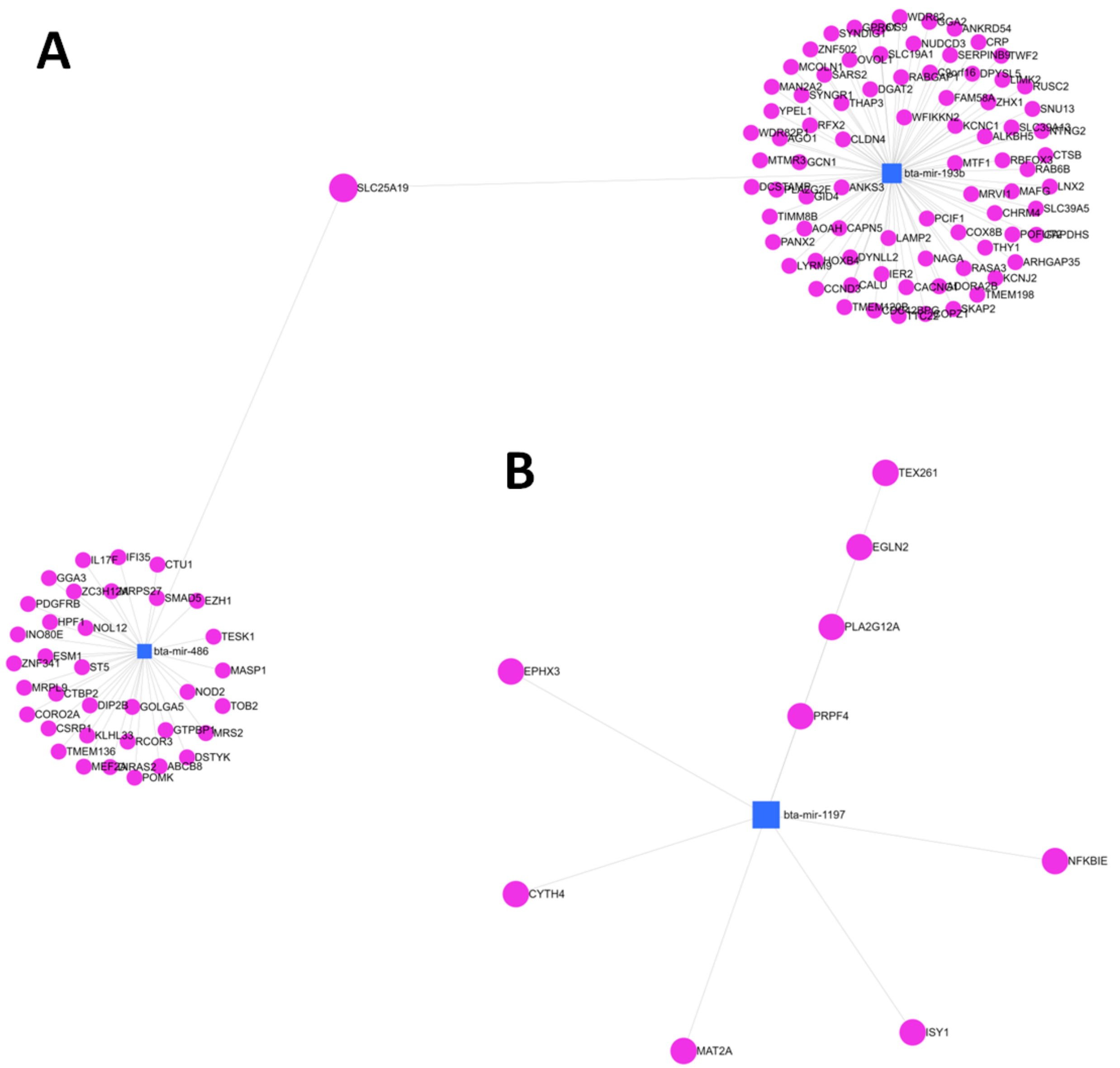

Supplemental Figure S1. Predicted potential target genes of the (**A**) upregulated and (**B**) downregulated differentially expressed (DE)-miRNA in subcutaneous adipose tissue of high-conditioned versus moderate-conditioned dairy cows on d -49 relative to calving. The DE-miRNA-target gene network was constructed using miRNet. The blue squares represent the (A) upregulated and (B) downregulated DE-miRNA, and the red circles represent the potential target genes.

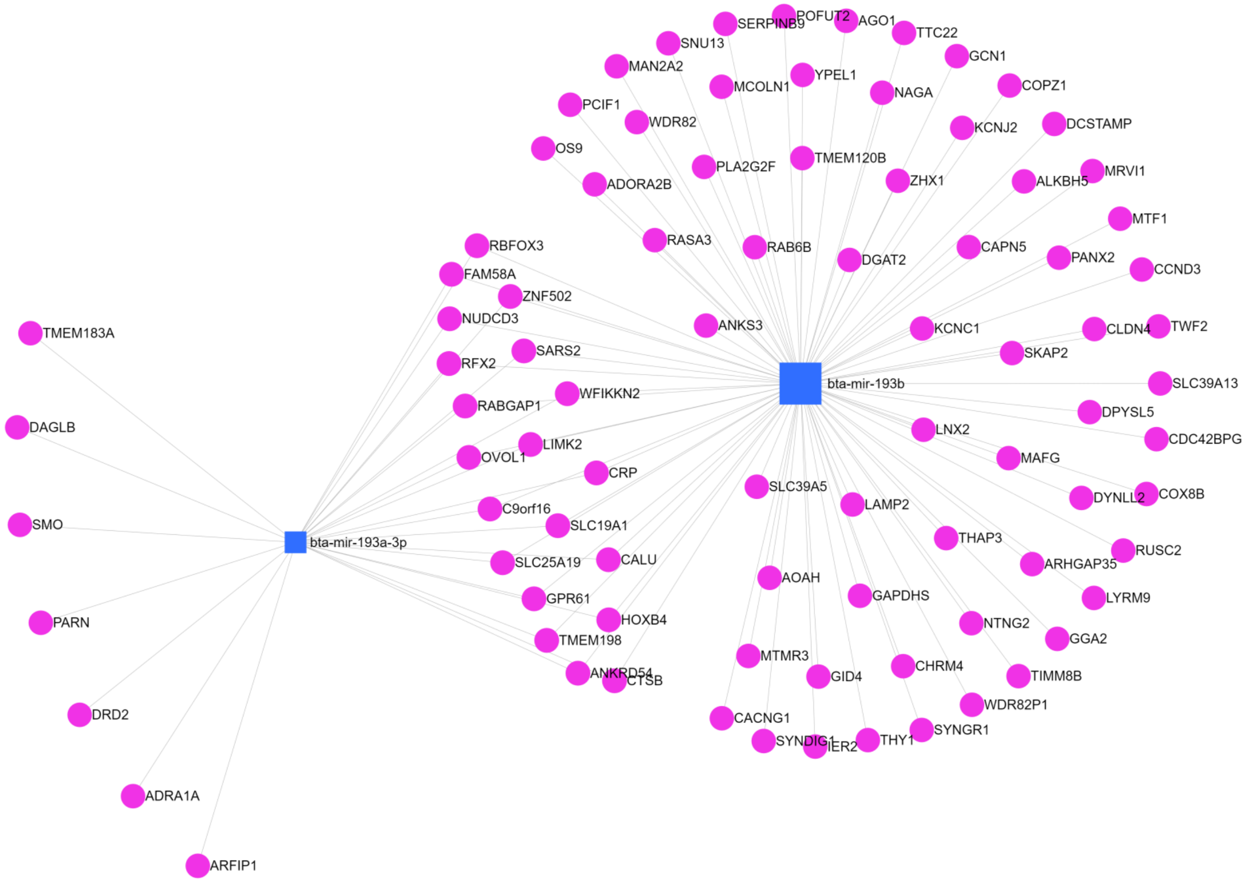

Supplemental Figure S2. (**A**) Predicted potential target genes of the upregulated differentially expressed (DE)-miRNA in subcutaneous adipose tissue of high-conditioned versus moderate-conditioned dairy cows on d 21 relative to calving. The DE-miRNA-target gene network was constructed using miRNet. The blue squares represent the upregulated DE-miRNA, and the red circles represent the potential target genes.

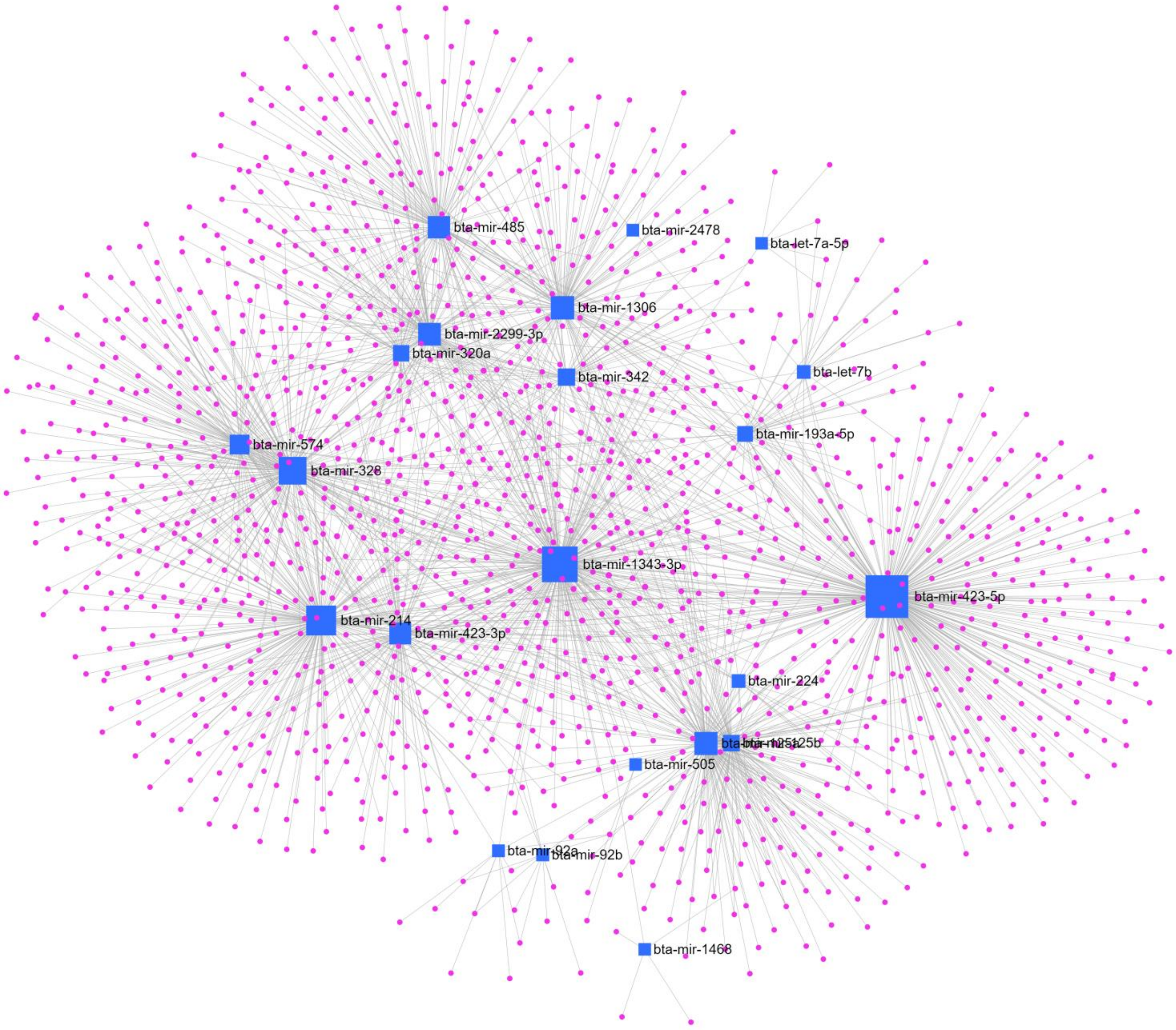

Supplemental Figure S3. Predicted potential target genes of the downregulated differentially expressed (DE)-miRNA in subcutaneous adipose tissue of high-conditioned versus moderate-conditioned dairy cows on d 21 relative to calving. The DE-miRNA-target gene network was constructed using miRNet. The blue squares represent the upregulated DE-miRNA, and the red circles represent the potential target genes. A list of potential target genes of downregulated DE-miRNA is given in Supplemental Excel File S1.
