# Supplementary material for: MicroRNA profiling of subcutaneous adipose tissue in periparturient dairy cows at high or moderate body condition
Source: Sci Rep. 2022 Aug 30;12:14748. doi: 10.1038/s41598-022-18956-5 (PMC9427980; doi:10.1038/s41598-022-18956-5)
Supplement: Supplementary file 3 — Supplementary Information 3. [file 41598_2022_18956_MOESM3_ESM.pdf]

Supplemental Table S1. Number of cows affected by clinical conditions occurring from calving to the first 6 weeks after calving in high (HBCS) and moderate-conditioned (MBCS) dairy cows.

	HBCS (n = 19)		MBCS (n = 19)	
Clinical condition	No. cows	No. cases	No. cows	No. cases
Mastitis	8	16	6	11
Ketosis	4	5	2	2
Milk fever	4	5	2	4
Locomotion	7	8	3	4
Retained fetal membranes/endometritis	1	1	2	2
Other conditions[1]	3	4	3	3
Total clinical conditions	27	39	15	26

[1] dystocia, inflammation of the conjunctiva of the eye

Supplemental Table S2. List of the predicted target genes of upregulated differentially expressed-miRNA that were enriched in selected relevant pathways in high-conditioned versus normal-conditioned cows on d 21 relative to calving.

Lysosome	Peroxisome	TNF signaling pathway	JAK-STAT signaling pathway
GGA2	ACSL5	FOS	IL10RA
MCOLN1	PXMP2	EDN1	IL10
LAMP2	ABCD4	SOCS3	SOCS3
NAGA	PEX11G	IL6	CCND3
CTSC		TNF	IL6
CTSB			IL10RB
PPT1			

Supplemental Table S3. List of the predicted target genes of downregulated differentially expressed-miRNA that were enriched in selected relevant pathways in high-conditioned versus normal-conditioned cows on d 21 relative to calving.

EGFR tyrosine kinase inhibitor resistance	Insulin resistance	HIF-1 signaling pathway	Focal adhesion	Autophagy	Ras signaling pathway	MAPK signaling pathway	PI3K-Akt signaling pathway
PLCG2	PTPA	PLCG2	THBS3	DDIT4	MRAS	NFATC1	THBS3
PDGFRB	PYGB	VEGFA	PDGFRB	MTMR3	PLCG2	MRAS	DDIT4
VEGFA	TBC1D4	ENO3	VEGFA	MRAS	KIT	MAPK8IP3	CASP9
PDGFRA	PRKAB1	PFKFB3	PDGFRA	RAB33B	PDGFRB	KIT	KIT
RPS6KB2	PTPN1	RPS6KB2	MAPK8	MAP1LC3A	STK4	PDGFRB	PDGFRB
EIF4E2	IKBKB	SLC2A1	ITGA11	CTSD	VEGFA	GADD45G	CDK2
SHC3	MAPK8	EIF4E2	PAK1	MAPK8	ZAP70	STK4	THEM4
PRKCG	RPS6KB2	HK1	FLNA	RPS6KB2	PDGFRA	DUSP3	VEGFA
FGFR2	SLC2A1	ENO1	SHC3	ATG4D	IKBKB	MAP3K11	PPP2R5D
SHC2	G6PC1	PRKCG	PPP1CA	ZFYVE1	GNG7	VEGFA	PDGFRA
MAPK3	CREB3	LTBR	COL4A1	STK11	MAPK8	PDGFRA	IKBKB
AKT1	PPP1CA	HMOX1	PRKCG	GABARAPL1	PAK1	CACNG1	GNG7
SHC1	RPS6KA1	MAPK3	PGF	WIPI2	RASSF5	IKBKB	ITGA11
PRKCB	SLC27A4	AKT1	ITGA5	RAB7B	SHC3	MAPK8	ATF6B
STAT3	CREB3L2	CAMK2A	ARHGAP35	ATG4B	CSF1R	DUSP9	RPS6KB2
PDGFB	AKT1	PRKCB	SHC2	MAPK3	IGF2	CACNA1D	G6PC1
EIF4EBP1	PRKCB	STAT3	MAPK3	ATG13	PRKCG	PAK1	EIF4E2
NRAS	STAT3	EIF4EBP1	AKT1	AKT1	PGF	FLNA	CREB3
	TNF	RBX1	AKT3	AKT3	FGFR2	TGFB3	STK11
	GYS1		PPP1R12C	DAPK3	FGFR1	CSF1R	CSF1R
			ACTN1	ATG9A	SHC2	IGF2	COL4A1
			ITGB7	NRAS	MAPK3	PRKCG	IGF2
			PAK6		AKT1	PGF	PGF
			COL6A2		AKT3	FGFR2	ITGA5
			ITGA3		PAK6	RPS6KA1	FGFR2
			SHC1		SHC1	CD14	IFNAR2
			PRKCB		RASA3	FGFR1	FGFR1
			PDGFB		PRKCB	MAPK3	CREB3L2
			VCL		NGFR	MAPKAPK3	MAPK3
			ACTB		PDGFB	AKT1	AKT1
					CALM1	MAPK12	AKT3
					RALB	TGFBR2	ITGB7
					FASLG	PRKCB	COL6A2
					EFNA3	NGFR	ITGA3
					NRAS	CACNG6	PPP2R5C
					PLA2G2F	PDGFB	NGFR
						CACNB3	CSF3
						TNF	PDGFB
						MAPK8IP2	EIF4EBP1
						FASLG	FASLG
						EFNA3	EFNA3
						NRAS	GYS1
						CACNG4	NRAS
